# Clinical Tolerogenic Dendritic Cells: Exploring Therapeutic Impact on Human Autoimmune Disease

**DOI:** 10.3389/fimmu.2017.01279

**Published:** 2017-10-12

**Authors:** Brett Eugene Phillips, Yesica Garciafigueroa, Massimo Trucco, Nick Giannoukakis

**Affiliations:** ^1^Allegheny Health Network Institute of Cellular Therapeutics, Allegheny General Hospital, Pittsburgh, PA, United States; ^2^Department of Biological Sciences, Carnegie Mellon University, Pittsburgh, PA, United States

**Keywords:** tolerogenic dendritic cells, autoimmune disease, autoimmunity, clinical therapeutics, type 1 diabetes, Crohn’s disease, rheumatoid arthritis, multiple sclerosis

## Abstract

Tolerogenic dendritic cell (tDC)-based clinical trials for the treatment of autoimmune diseases are now a reality. Clinical trials are currently exploring the effectiveness of tDC to treat autoimmune diseases of type 1 diabetes mellitus, rheumatoid arthritis, multiple sclerosis (MS), and Crohn’s disease. This review will address tDC employed in current clinical trials, focusing on cell characteristics, mechanisms of action, and clinical findings. To date, the publicly reported human trials using tDC indicate that regulatory lymphocytes (largely Foxp3+ T-regulatory cell and, in one trial, B-regulatory cells) are, for the most part, increased in frequency in the circulation. Other than this observation, there are significant differences in the major phenotypes of the tDC. These differences may affect the outcome in efficacy of recently launched and impending phase II trials. Recent efforts to establish a catalog listing where tDC converge and diverge in phenotype and functional outcome are an important first step toward understanding core mechanisms of action and critical “musts” for tDC to be therapeutically successful. In our view, the most critical parameter to efficacy is *in vivo* stability of the tolerogenic activity over phenotype. As such, methods that generate tDC that can induce and stably maintain immune hyporesponsiveness to allo- or disease-specific autoantigens in the presence of powerful pro-inflammatory signals are those that will fare better in primary endpoints in phase II clinical trials (e.g., disease improvement, preservation of autoimmunity-targeted tissue, allograft survival). We propose that pre-treatment phenotypes of tDC in the absence of functional stability are of secondary value especially as such phenotypes can dramatically change following administration, especially under dynamic changes in the inflammatory state of the patient. Furthermore, understanding the outcomes of different methods of cell delivery and sites of delivery on functional outcomes, as well as quality control variability in the functional outcomes resulting from the various approaches of generating tDC for clinical use, will inform more standardized *ex vivo* generation methods. An understanding of these similarities and differences, with a reference point the large number of naturally occurring tDC populations with different immune profiles described in the literature, could explain some of the expected and unanticipated outcomes of emerging tDC clinical trials.

## Introduction

Autoimmune diseases are characterized by the loss of tolerance to self-antigens resulting in the immune system targeting a wide range of tissues leading to impaired function, tissue eradication, and clinical morbidity and mortality. Many of the current therapeutics manage symptoms of a general inflammatory state, even if they target specific molecules on inflammatory cells and/or their secreted products (e.g., immunokines). Autoimmunity requires ongoing, often lifelong treatment. While systemic immunosuppressives are still the mainstay of treating most autoimmune conditions, biologic-based immunotherapies selectively targeting specific molecules and pathways have become part of the treatment approach, although their side effects often cause more problems than they intend to solve. Cell therapy has been a sought after alternative, or adjunctive approach for at least two decades, since the discovery of tolerogenic dendritic cells (tDC) and with the more recent characterization of T-regulatory cells (Tregs) (1–9). In this review, we will summarize the current tDC-based clinical trials, as well as those that are planned for the treatment of autoimmune diseases. We will point out the common features and the common mechanisms that they share in their functional outcomes and also highlight some key questions that remain to be answered to ensure that these cells remain stably tolerogenic *in vivo*.

Dendritic cells are considered to be the body’s “professional” antigen-presenting cells (10–15) and they regulate adaptive immunity and maintain immune homeostasis in the periphery (16). When DC express low levels of surface proteins, collectively referred to as co-stimulation molecules (e.g., CD86, CD40, OX-40), produce little to no IL-12p70, and exhibit low nuclear factor kappa-light-chain-enhancer of activated B-cells (NF-κB) transactivational activity, they are referred to as “immature” (17–20). DC reside inside peripheral tissues throughout the body in this state under normal conditions and they acquire either draining tissue antigens or migrate through the tissues and stromal structures, acquiring antigens through phagoendocytic mechanisms (e.g., trogocytosis) (21–24). They remain as immature cells until the time they encounter a pro-inflammatory environment. When antigens are acquired in an environment of inflammation, such as an infection, DC undergo a series of maturation steps that increase the expression and cell surface level of major histocompatibility complex (MHC) class II molecules for antigen presentation concurrent with the upregulation of co-stimulation molecules, and production of IL-12p70 that together act in concert to stimulate the division and functional polarization of T-cells (25–28). Mature DC do this consequent to their accumulation inside the lymph nodes or lymphoid structures that drain the site from which they acquired the antigens. There, inside the lymphoid organs, they present those antigens to the T-cell receptor on naïve T-cells. A series of secondary interactions with co-stimulatory molecules fully activate T-cells (29, 30). Antigens presented in this fashion are typically foreign, but in autoimmune diseases self-antigens are presented to potentially autoreactive T-cells leading to targeted destruction of tissues (31).

Dendritic cells that acquire antigens but do not receive signals to undergo maturation maintain their immature state and can also present antigens to naïve T-cells in secondary lymphoid organs. In the absence of co-stimulation, these DC usually induce a state of anergy in target T-cells leading to peripheral tolerance. Immature DC further facilitate peripheral immune tolerance by maintaining populations of naturally occurring thymic Tregs and/or induce naïve T-cells to differentiate into peripheral Tregs as they also shift differentiated T-helper (T_h_) cell phenotypic and functional activity balance toward cell populations representing the T_h2_ side (1, 32–37). This outcome is usually a consequence of IL-10 gene activation and immunokine production by the DC instead of IL-12p70, which augments the T_h2_ subpopulation and, in a paracrine feedback manner, inhibits DC maturation (38). While autologous Tregs therapy is an alternative approach to treating autoimmune disease, it is limited by questionable stability of the administered cells *in vivo* (39–41), polyclonality (42–44), and concerns about systemic dissemination of the cells since they are administered intravenously. From a manufacturing perspective, the volume of blood currently needed to generate an injectable cell product (approximately 400 ml per patient) can be prohibitive. Instead, the advantages of tDC lie in their multiple mechanisms to treat disease that involve anergy of autoreactive T-cells, activation of different regulatory lymphocyte populations, dynamic antigen acquisition *in vivo* and presentation to autoreactive T-cells to induce hyporesponsiveness, and migration into lymphoid regions draining the disease target. Over the past 20 years, much research has been invested toward the characterization of these immature DC and into methods that can generate them *in vitro* from hematopoietic progenitors and maintain them stably in an immature state capable of possibly restoring tolerance *in vivo* in autoimmune diseases (2, 9, 17, 45–52).

## Type 1 Diabetes (T1D) Mellitus

Type 1 diabetes is a disease that leads to the progressive loss of pancreatic beta cells and insulin production. Insulin replacement is the only and current gold standard of therapy, but even rigorous control of blood glucose levels fails to prevent the development of diabetic complications (53). These complications include neuropathy, nephropathy, vision loss, and cardiovascular disease which are associated with high morbidity and mortality (54). Devices for the delivery of insulin may mimic how insulin is secreted and could potentially reduce diabetes-related complications (55, 56), but they do not address the underlying autoimmune pathology, nor is insulin release fully coupled to second-to-second fluctuating glucose levels. Autoimmunity suppression is also a hurdle for the implementation of islet transplants that, while reducing or delaying the clinical outcome of complications, would come under the same rejection by leukocytes even with the application of drugs to prevent tissue rejection (57–60).

Tolerogenic dendritic cells are a potential therapy for the treatment of new onset T1D to prevent the further destruction of pancreatic beta cells. Loss of beta cell mass can reach 80% by time of diagnosis (61), making the therapeutic window small, but feasible. The first tDC clinical trial for the treatment of autoimmune disease was for T1D (clinicaltrials.gov identifier: NCT00445913) (62). Monocytes were isolated by leukapheresis and grown *ex vivo* in the presence of granulocyte macrophage colony-stimulating factor (GM-CSF) and interleukin-4 (IL-4) for 6 days. Cells for the treatment arm of the study were cultured with a mixture of antisense oligonucleotides targeting the primary transcripts of the CD40, CD80, and CD86 co-stimulatory molecules at a concentration of 3.3 µM each oligonucleotide. Cells proven to exhibit reduced expression of these co-stimulation proteins (by flow cytometry) and passing the viability and sterility screen were given to patients in four treatments of 1.0 × 10^7^ cells, where each round of administration was 2 weeks apart. Each round of treatment was divided into four intradermal injection sites proximal to the expected anatomical location of the pancreas in an effort to enhance DC migration to the pancreatic and peri-pancreatic lymph nodes, based on known and suspected lymphatic drainage fields. All tDC were from thawed cryopreserved cell stocks. Ten patients were recruited for the phase I study; 3 patients in the control arm and 7 in the tDC treatment arm. Safety for patients was assessed in-trial (12 months).

The tDC were well tolerated without any adverse events noted. Two novel findings resulted from the study. First, the tDC-treated arm displayed a transient elevation of B220+ CD11c− B cells that, during the study, appeared to contain a subpopulation of B-regulatory cells (Bregs). The presence of Bregs and the effect of the tDC on their generation was demonstrated in a follow-up study (63). The second finding was that, in 4/7 patients who were insulin C-peptide negative at baseline, there was a conversion to C-peptide positivity to sub-physiological concentrations in 3/7, but to physiological levels in one patient, during the tDC administration cycle. C-peptide is the cleavage product of proinsulin as it matures into insulin during its biosynthesis and secretory phases inside the pancreatic beta cells and is used as a surrogate marker for functional beta cells (64). However, this trial’s intent was to assess safety of the tDC and in spite of these findings, there was no attempt to determine if insulin dosage could be adjusted. Patients recruited in this study were diabetic and insulin-requiring for a minimum of 5 years and, therefore, should not have been expected to harbor significant residual beta cell mass. The emergence of detectable C-peptide during the tDC treatment cycle suggests restoration of insulin production from remaining islets or possible new islet formation. There were no significant differences in other measurements between control and tDC treatment arms (e.g., in cytokine serum concentrations or cell population number other than Bregs), even though a subtle, albeit statistically insignificant increase in Tregs number were detected in tDC-treated patients.

## Rheumatoid Arthritis (RA)

Rheumatoid arthritis is an inflammatory disease that targets the cartilage of the joint articulations, with the highest rate of occurrence in small joints of the hands and feet (65). Chronic inflammation further results in loss of bone mass, tendon inflammation, and rupture associated with airway and cardiovascular complications (66). Current treatment strategies require continuous treatment with anti-inflammatory drugs and biologics. These, however, fail to maintain remission over the life of the disease. With an RA global incidence rate as high as 1% of adults (67), there is a large patient population that could benefit from tDC therapeutics.

### Rheumavax RA Study

The first-in-human trial for the treatment of RA generated tDC by NF-κB inhibition (clinicaltrials.gov identifier: NCT00396812) (68). The transcription factor NF-κB controls gene expression of genes involved in many pro-inflammatory pathways, making it a target of choice for anti-inflammatory drugs (69). Inhibition of NF-κB prevents DC maturation, reduces the expression of CD40 and human leukocyte antigen–antigen D related (HLA-DR, a class II MHC molecule), and confers tolerogenic properties to DC including induction of T-cell anergy (70, 71). Isolated monocytes were grown in the presence of IL-4, GM-CSF, and 2–2.5 µM of the NF-κB inhibitor Bay 11-7082 for 48 h. DC were further prepared in a 3-h exposure to citrullinated peptides of aggrecan, vimentin, collagen type II, and a and b fibrinogen which are putative RA autoantigens (72) as anti-citrullinated protein antibodies are found in 50–80% of patients over the lifetime of the disease (65). Preloading tDC with disease-specific autoantigens increases the likelihood of their presentation to T-cells inside the inflamed joint-draining lymph nodes, thus disrupting the cycle of autoreactive T-cell activation. The resulting generated tDC displayed a 5% reduction in the mean fluorescence intensity (flow cytometric measurement) of CD40 and a 17% reduction in HLA-DR when assessed by flow cytometry (68). Patients were given a single intradermal injection of 1.0 × 10^6^ or 5.0 × 10^6^ tDC.

The treatment was generally well tolerated and deemed safe. General trends indicated a 25% decrease in pro-inflammatory T-cells (CD4+ CD25+ CD127+) and 25% increase in anti-inflammatory Treg (CD4+ CD25+ high CD127−) within 1 month of treatment. Circulating levels of the inflammation marker C-reactive protein (CRP) were significantly decreased in patients receiving the high cellular dose. Similarly, cytokine expression profiles for IL-15, CXCL1, CXCL11, IL-29, and peptide YY were reduced in patients receiving the high dose Rheumavax treatment. Disease activity scores 28 (DAS28), a common metric used for the evaluation RA severity, were decreased in a portion of the patients.

### Newcastle University RA Study

The second RA tDC trial was conducted at the University of Newcastle and used dexamethasone (Dex) and vitamin D3 for tDC generation (clinicaltrials.gov identifier: NCT01352858) (73). Dex is a synthetic glucocorticoid that has a range of powerful anti-inflammatory effects in the clinical setting (74). Dex inhibits the NF-κB pathway through a number of mechanisms. The most prominent includes increased nuclear factor kappa-light-chain-enhancer of activated B-cells inhibitor, alpha (IκBα) expression which binds and retains the RelA subunit of NF-κB inside the cytoplasm preventing transcriptional activities inside the nucleus (75, 76). tDC grown in the presence of Dex exhibit decreased expression of co-stimulation proteins CD40 and CD86 and the DC maturation marker CD83, along with decreased class II MHC expression and IL-12p70 production (71, 77–79). These tDC produced high concentrations of the immunosuppressive IL-10 immunokine (80). Similar alterations in DC surface and cytokine expression profiles can also result with vitamin D3 treatment *in vitro* (81–83). Interestingly, vitamin D3 deficiency is associated with RA and poorer clinical outcomes (84, 85). Generation of tDC with both Dex and vitamin D3 has an additive effect on IL-10 production levels (26, 86).

In this trial, monocytes were isolated by density centrifugation followed by microbead selection of CD14 expressing cells. Monocytes were grown in culture for 7 days in the presence of 50 ng/ml IL-4 and 50 ng/ml GM-CSF; with the addition of 1 µM Dex on day 3 and day 6, 0.1 nM vitamin D3 on day 6, and 1.0 µg/ml monophyosphoryl lipid a (MPA) on day 6. Cells were then cocultured with synovial fluid collected from inflamed joints of study patients allowing for unique autoantigen loading specific to each patient. The patient-specific tDC were characterized with reduced CD40, CD83 surface levels and decreased IL-12p70 production while maintaining high concentrations of secreted IL-10 (73, 77). After tDC passed sterility testing, patients received a single injection of saline, 1.0 × 10^6^, 3.0 × 10^6^, or 1.0 × 10^7^ cells into the affected knee joint. The treatment was deemed safe with no worsening knee flares and a reduction in symptoms of patients treated with the high dose. Peripheral blood immune T-cell populations (CD4+ IL-10+, CD4+ FoxP3+, CD4+ IFNγ+, CD4+ IL-17+) and cytokines production levels [IL-10, interferon gamma (IFNγ), IL-17, IL-6, tumor necrosis factor alpha (TNFα)] were unaltered.

## Crohn’s Disease

Crohn’s disease is an autoimmune disease of the gastrointestinal (GI) tract that can affect tissues from the mouth to the anus (87). Common symptoms include abdominal pain, bloody diarrhea, inflammation, weight loss, and bowel blockage (87, 88). Current treatments are designed to manage the symptoms, but disease flare-ups are common. There are no specific therapies against the underlying autoimmunity. A single phase I clinical trial has been reported as completed, testing the safety of tDC (European Clinical Trials Database number 2007-003469-42) (89).

The immunologic space of the intestine is exposed to a high number of foreign antigens provided by intestinal flora. The breakdown of immune control is mediated by inappropriate activation of T_h1_ and Th17 cells and the loss of retinaldehyde dehydrogenase (RALDH)-positive DC. This DC subpopulation may be the reason vitamin A was incorporated into the tDC generation process for this trial. Vitamin A deficiency is prevalent in patients with Crohn’s disease and correlates with disease severity (90). Conventional CD103+ CD11b+ intestinal DC convert vitamin A to retinoic acid through expression of RALDH which is atypical of DC found in draining lymph nodes (91). DC-generated retinoic acid maintains tolerance to GI tract cells and tissues through enhanced CD4+ T cell recruitment to the intestine and differentiation into FoxP3+ T-cells and T_h17_ from existing CD4+ T-cell populations (1, 26, 92, 93). Furthermore, generation of retinoic acid-producing DC naturally inside the disease-affected tissues as a consequence of administration of retinoic acid-producing tDC could establish an ongoing “feed forward” type of tDC generation and stabilization cycle in the patient’s intestinal epithelial cells. This clinical trial relies on proximal tDC delivery, but mentions that future methods may switch to direct delivery of tDC into intestinal lesions (89).

For the generation of tDC in this trial, monocytes were obtained by leukapheresis. Cells were cultured in 500 UI/ml IL-4 and 800 UI/ml of GM-CSF for 7 days; 1 µM of Dex and 1 nM vitamin A starting on day 3; and the cytokines IL-1β, IL-6, TNFα, and prostaglandin E2 for the final day (89, 94). The cell products exhibited elevated CD80 and CD86, and low CD83 expression. MERTK, a glucocorticoid-induced receptor that is prevalent in tDC was also expressed at high levels. Production of IL-10 was detected in the cells with no detectable IL-12p70 or IL-23 in the cell culture media. Allogenic mixed lymphocyte reactions performed in the presence of tDC resulted in low T-cell proliferation and IFNγ production. tDC were administered to Crohn’s patients by intraperitoneal injection in six different treatment arms based on the number of administered cells (2.0 × 10^6^, 5.0 × 10^6^, 1.0 × 10^7^) and number of injections (one dose or three doses spread out every 2 weeks). These tDC were well tolerated. One-third of the patients completing the study showed a clinical improvement based on a Crohn’s disease activity index. T_h1_ and Th17 cell populations were unchanged in numbers in circulation, but there was a significant increase in circulating Tregs (CD4+ CD25+ Foxp3+) 12 weeks after injection when compared to baseline. Isolated T-cells stimulated with CD3 antibody secreted less IFNγ suggesting that the tDC had established some form of immune hyporesponsiveness in the patients.

A second clinical trial has been initiated for Crohn’s disease using tDC (clinicaltrials.gov identifier: NCT02622763); however, at this time very few details are known about the methods of tDC generation.

## Multiple Sclerosis (MS)

Multiple sclerosis is an autoimmune disease that results in the demyelination of neurons in the central nervous system as well as in the peripheral nervous system. Demyelination is mediated by autoreactive T-cells activated by self-antigen presentation by DC. A number of drugs and biologics are being used to inhibit various immune pathways (95), and tDC are currently being used in two phase I clinical trials. To date, the results of these trials have not been yet published. The first trial (clinicaltrials.gov identifier: NCT02283671) utilizes tDC generated in the presence of IL-4, GM-CSF, and Dex. These cells are pre-loaded with myelin self-peptides and are administered intravenously in three injections each 2 weeks apart. The second trial (clinicaltrials.gov identifier: NCT02618902) considers tDC generated in the presence of vitamin D3 and similarly preloads cells with myelin self-peptides. Patients will receive 5.0 × 10^6^, 1.0 × 10^7^, or 1.5 × 10^7^ cells spread over five intradermal injection sites in the subclavicular region. This will be the highest administered dose of tDC described in current tDC clinical trials, which was probably informed by the safety reports of previous tDC trials. Similar to retinoic acid, vitamin D levels are lower in patients with MS than healthy individuals. Relapse of MS symptoms are also associated with lower vitamin D levels when compared to MS patients that are currently in intermission (96, 97). Generation of tDC from healthy and MS patients in the presence of vitamin D3 results in reduced tDC IL-12 and IL-23 cytokine secretion, inhibited maturation, and increased CD83/decreased CD80 cell surface expression (95).

## Discussion

Tolerogenic dendritic cells have, or are currently being tested in phase I clinical trials for T1D, RA, MS, and Crohn’s disease, with additional considerations aiming at lupus (98) and facilitating allogeneic tissue and organ transplantation (9, 99–101). tDC generation relies on the use of IL-4 and GM-CSF to differentiate monocyte progenitors, and these cytokines remain the central feature shared among all the tDC generation methods. The differences, however, lie in the additional factors added in the cell cultures from the time of monocyte seeding to the last changes in media prior to tDC harvest (e.g., putative autoantigens, vitamin D3, immunosuppressives like Dex and NF-κB inhibitors, antisense oligonucleotides targeting co-stimulation) (Table 1). To what extent these conditions change cellular effectiveness and mechanism of action of tDC to confer their potentially beneficial effects is unclear at present. Nevertheless, most tDC share one mechanistic feature: increased regulatory lymphocytes (e.g., Foxp3+ Tregs and Bregs) in the peripheral blood of patients during administration (62, 68, 89).

**Table 1:** A comparison of current tolerogenic dendritic cells (tDC) and their clinical application for completed and ongoing clinical trials.

Disease/trial	Diabetes (62) Pittsburgh	Rheumatoid arthritis (RA) Rheumavax (68)	RA Newcastle University (73)	Crohn’s disease (89)	Multiple sclerosis (MS)	MS
Clinical Trial ID	NCT00445913	NCT00396812	NCT01352858	2007-003469-42	NCT02283671	NCT02618902

Cell generation						
NF-κB inhibitor	–	BAY 11-7082	Dexamethasone (Dex)	Dex	Dex	–
Vitamins	–	–	Vitamin D3	Vitamin A	–	Vitamin D3
Stimulation	–	–	Monophyosphoryl lipid A	Cytokines	Unpublished	Unpublished
Antigens	–	Citrullinated peptides	Synovial fluid	–	Myelin peptides	Myelin peptides
Other	With or without Antisense CD40, CD80, CD86					

Cell characterization					Unpublished	Unpublished
Sterile/viable	Passed	Passed	Passed	Passed		
CD40	X	↓	=	X		
CD80	X	↓	X	↑		
CD83	X	X	↓	↓		
CD86	X	=	↑	↑		
IL-10	X	X	↑	↑		
IL-12	↓	X	↓	↓		

Therapeutics						
Cell number	1.0 × 10^7^	1.0 or 5.0 × 10^6^	1.0, 3.0, or 10.0 × 10^6^	2.0, 5.0, or 10.0 × 10^6^	Unpublished	5.0, 10.0, or 15.0 × 10^6^
Injection number	4 injections	1 injection	1 injection	1 injection	Unpublished	5 injections
Injection site	Intradermal	Intradermal	Knee joint	Intraperitoneal	Intravenous	Intradermal
Dose number	4, 2 weeks apart	1	1	1 or 3, 2 weeks apart	3, 2 weeks apart	Unpublished

Research outcomes						
Tolerated	Tolerated	Tolerated	Tolerated	Tolerated	Unpublished	Unpublished
T-regulatory cell	↑	↑	=	↑		
Plasma cytokines	↑IL-4, IL-10	↓IL-15, IL-29	=	X		

Disease outcomes	Elevated C-peptide	↓ CRP	Tolerated	Improved Crohn’s disease activity index	Unpublished	Unpublished
	B-regulatory cells population	↓ DAS28		Reduced IFNγ after *ex vivo* CD3 stimulation		

*Cell generation displays the reagents used in tDC preparation (not including shared IL-4 and GM-CSF components) and cell characterization displays surface markers and cytokine secretion profiles of pre-injected cells. Table entries marked as “X” are values that were not assessed within a given trial. Arrows indicate a change for a given value, but were not present in all patients within a study, exist at specific time points that may not be maintained for the duration of the study, or failed to reach significance in some studies. MS studies are still underway and unpublished. The information provided derives from clinicaltrial.gov entries for these registered clinical trials and is current as of August 29, 2017*.

Another difference among the tDC used in clinical trials lies in the dose level administered and site of cell delivery in the body. This last point is relevant in the mechanism of tDC since affected tissues and focal points of inflammation differ among autoimmune diseases. The majority of tDC clinical trials to date deliver tDC proximal to the site of inflammation, with the desired goal of tDC migration into the local draining lymph node. Draining lymph nodes adjacent to the site of inflammation have a great preponderance of activated self-reactive T-cell populations to target for anergy (102). The clinical studies described so far have used between 1 and 5 injection sites per cell treatment cycle, targeting one or more pertinent lymph nodes such as the cervical lymph nodes in the MS study (clinicaltrials.gov identifier: NCT02618902). An alternative approach is to directly introduce tDC into the site of inflammation. Direct administration of tDC to lesion sites in Crohn’s disease was not attempted but suggested for future study. This would address a different mode of action, where the vitamin A-generated tDC could potentially restore a lost intestinal subpopulation of tDC specific to Crohn’s disease. Targeting “niche” tDC populations may require the need for the generation of tDC with more restricted immunosuppressive phenotypes. While the Newcastle University RA study introduced tDC directly at the site of inflammation, the intended goal was still for the migration of tDC to local draining lymph nodes. Even though the technique is more invasive than intradermal administration, the introduction of tDC producing IL-10 may have the added benefit of local immunosuppression at the point of inflammation. This consideration is balanced by the possibility that local inflammatory conditions may alter the introduced tDC phenotype to a more pro-inflammatory state.

Autoimmune diseases each have their own unique autoantigens and associated self-reactive T-cell populations. Preloading tDC with specific disease antigens enhances their ability to directly interact and inactivate self-reactive T-cells that cause tissue damage. The Rheumavax RA study loaded tDC with citrullinated peptides identified from 70% of RA patients who exhibit auto-antibodies to these targets. To further this strategy, they selected patients with high risk HLA alleles that have a strong association with citrullinated auto-antibody positivity. Unfortunately not all patients display uniform self-antigens for a given disease. T1D, for example, is associated with a range of self-antigens and auto-antibodies that are differentially expressed among patients and at different points during the disease. Even though there seems to be a general consensus about insulin and GAD65-derived peptide-pulsing tDC for T1D, antigen spreading that has occurred at the time of clinical disease may limit the autoreactive T-cell populations targetable, whereas other “late-antigen”-specific T-cells may in fact be driving autoimmunity after clinical onset. In an elegant study designed by the Newcastle University group, the RA trial overcame this potential limitation by collecting synovial fluid from inflamed joints of each patient. tDC were pre-exposed to autologous synovial fluid for antigen collection, and then given an additional chance to acquire patient-specific autoantigens through direct administration of tDC to the site of inflammation. If initial tDC therapeutics trials are successful, further studies may wish to look at the effectiveness of matching patient autoantigens despite the potential increase in manufacturing and quality control costs.

Currently, only four of the discussed clinical trials have been completed with reported outcomes (62, 68, 73, 89). Despite the different approaches used to generate the tDC in these trials, NF-κB inhibition is the central feature of 3 of these studies, with one study also including the use of vitamin D3. Generation of tDC with either NF-κB inhibitors or vitamin D3 promotes immature DC phenotypes with an additive effect when using both agents. The Newcastle University RA (Dex + Vit D3) and the Crohn’s disease (Dex) trials both reported decreased CD83 expression, high CD86 expression, decreased IL-12 secretion, and elevated IL-10 secretion in their tDC products suggesting a possible tDC shared phenotype. Pre-activation of tDC with cytokines or lipid immune mediators is also shared between these two protocols. The *Rheumavax* RA (BAY 11-7082) study measured different parameters, but did report a divergent decrease in CD80 surface levels. In contrast, the T1D clinical trial directly intervened to reduce and maintain stably reduced co-stimulatory molecules CD40, CD80, and CD86 without the use of an NF-κB inhibitor, but other than demonstrating low IL-12 concentrations during stimulation *in vitro*, it did not further characterize the generated tDC beyond purity and sterility. Without full characterization of, at least, the immune phenotypes and functional immune activities, it will be difficult to compare the mechanisms of action among the different tDC to functionally identify their points of intersection (e.g., do all tDC promote Tregs, and how? Are key immunoregulatory immunokines produced by all tDC, and/or what are the immunokines that tDC elicit in common among the different T_h_ cell populations?). The difficulty in comparing the characteristics of different clinical tDC does suggest the need for an uniform set of metrics for their description. This was the focus of the minimum information about tolerogenic antigen-presenting cells (103) initiative whose authors included members from a number of the completed and ongoing clinical trials.

Much of the current divergence in tDC phenotype and points of mechanistic intersection other than increased frequency of regulatory immune cells in the peripheral blood during treatment might also be due to the *ex vivo* upstream cell processing prior to the addition of GM-CSF/IL-4 (e.g., monocyte progenitors, contaminating granulocytes in the monocyte elutriation). An important question that needs to be addressed is the relevance of the tDC method and site of delivery (intravenous, subcutaneous, intradermal) on their effect and mechanism of action (direct or indirect) at the lymphoid organs draining the inflamed tissues and/or the autoimmunity target tissues proper. Finally, it is important to determine if freshly generated versus thawed cryopreserved tDC are functionally different *in vivo*. Considering the limitations and adverse events encountered using biologic agents and the need to move past systemically acting immunosuppressives, the well-tolerated safety profile of tDC across a range of dose levels and administration sites, along with the evidence of increased regulatory cell frequency *in vivo* during treatment, strongly argues in favor of their further development, characterization, and consideration to fundamentally change how autoimmune diseases are treated, directly addressing the immune imbalance and moving away from disease and symptom management.

## Author Contributions

BP and YG wrote the manuscript and MT and NG edited while adding additional insights. The final version was proofread and edited by NG.

## Conflict of Interest Statement

NG and MT hold equity in Diavacs Inc., which has licensed the intellectual property concerning the tolerogenic dendritic cells noted in the review under the type 1 diabetes clinical trial (also referred to as the “Pittsburgh tolerogenic dendritic cells”). The other authors do not have any conflicts of interest, real or potential. The handling editor declared a past co-authorship with the authors NG and MT.

## Abbreviations

APC, antigen-presenting cells; Bregs, B-regulatory cells; DAS28, disease activity scores 28; DC, dendritic cells; Dex, dexamethasone; GM-CSF, granulocyte macrophage colony-stimulating factor; HLA-DR, human leukocyte antigen-antigen D related; IFNγ, interferon gamma; IκBα, nuclear factor kappa-light-chain-enhancer of activated B-cells inhibitor, alpha; IL, interleukin; MHC, major histocompatibility complex; MITAP, minimum information about tolerogenic antigen-presenting cells; MPA, monophyosphoryl lipid A; NF-κB, nuclear factor kappa-light-chain-enhancer of activated B-cells; RA, rheumatoid arthritis; RALDH, retinaldehyde dehydrogenase; T1D, type 1 diabetes; TCR, T-cell receptor; tDC, tolerogenic dendritic cells; Th, T-helper cells (1,2, or 17); TNFα, tumor necrosis factor alpha; Tregs, T-regulatory cells.
